# Proteasomal Degradation of TRIM5α during Retrovirus Restriction

**DOI:** 10.1371/journal.ppat.1000074

**Published:** 2008-05-23

**Authors:** Christopher James Rold, Christopher Aiken

**Affiliations:** Department of Microbiology and Immunology, Vanderbilt University School of Medicine, Nashville, Tennessee, United States of America; Northwestern University, United States of America

## Abstract

The host protein TRIM5α inhibits retroviral infection at an early post-penetration stage by targeting the incoming viral capsid. While the detailed mechanism of restriction remains unclear, recent studies have implicated the activity of cellular proteasomes in the restriction of retroviral reverse transcription imposed by TRIM5α. Here, we show that TRIM5α is rapidly degraded upon encounter of a restriction-susceptible retroviral core. Inoculation of TRIM5α-expressing human 293T cells with a saturating level of HIV-1 particles resulted in accelerated degradation of the HIV-1-restrictive rhesus macaque TRIM5α protein but not the nonrestrictive human TRIM5α protein. Exposure of cells to HIV-1 also destabilized the owl monkey restriction factor TRIMCyp; this was prevented by addition of the inhibitor cyclosporin A and was not observed with an HIV-1 virus containing a mutation in the capsid protein that relieves restriction by TRIMCyp IVHIV. Likewise, human TRIM5α was rapidly degraded upon encounter of the restriction-sensitive N-tropic murine leukemia virus (N-MLV) but not the unrestricted B-MLV. Pretreatment of cells with proteasome inhibitors prevented the HIV-1-induced loss of both rhesus macaque TRIM5α and TRIMCyp proteins. We also detected degradation of endogenous TRIM5α in rhesus macaque cells following HIV-1 infection. We conclude that engagement of a restriction-sensitive retrovirus core results in TRIM5α degradation by a proteasome-dependent mechanism.

## Author Summary

Recent studies have identified several cellular proteins that restrict infection by a variety of retroviruses. One of these restriction factors, TRIM5α, is partially responsible for the differences in susceptibility of monkeys and humans to SIV and HIV-1, respectively. TRIM5α inhibits retrovirus infection soon after penetration into the target cell by associating with the viral protein CA, which forms the polymeric capsid shell of the viral core. Although the detailed mechanism of restriction is unknown, TRIM5α is postulated to alter the stability of the viral core, resulting in a failure to complete reverse transcription. The activity of cellular proteasomes, which are responsible for intracellular protein degradation, has also been implicated in TRIM5α-dependent attenuation of retroviral reverse transcription. In this study, we show that cellular TRIM5α is rapidly degraded in cells exposed to a restriction-sensitive retrovirus but not in cells infected with an unrestricted virus. Virus-induced degradation of TRIM5α was dependent on cellular proteasome activity, as inhibition with drugs blocking proteasome function also inhibited degradation of TRIM5α. These results provide additional support for a role of proteasomal degradation in TRIM5α-dependent retrovirus restriction and suggest a novel mechanism by which binding of TRIM5α to the viral capsid prevents infection.

## Introduction

Retroviruses exhibit a restricted host range due to the requirement for specific interactions between viral and host proteins to complete the viral life cycle. Also limiting retroviral tropism are several recently identified intracellular antiviral factors ([1]–[5]); reviewed in [6]–[10]). The prototypical restriction activity, Fv1, was first detected in the 1970s as differential susceptibility of inbred mice strains to the Friend leukemia virus [11]–[13]. Fv1 blocks infection of murine leukemia viruses (MLV) at a stage following fusion but prior to integration [14],[15]. The block to infection can be overcome at high multiplicities of infection (m.o.i.) or by pretreatment of target cells with non-infectious virus like particles (VLPs) [11],[16]. Susceptibility to Fv1 restriction is determined by the sequence of the viral capsid protein (CA) [17]–[19]. The gene encoding Fv1 was identified in 1996 by positional cloning [1]; yet the molecular mechanism by which Fv1 inhibits MLV infection remains poorly defined.

Recent investigations have identified additional restriction activities present in human and simian cells that govern the tropism of lentiviruses, including human and simian immunodeficiency viruses (HIV and SIV) [20]–[25]. Like Fv1, these restrictions target the incoming viral capsid [23], [25]–[27]. One factor, TRIM5α, is responsible for post-entry restriction of HIV-1 in many simian cell lines [3], [28]–[31]. Expression of the rhesus macaque TRIM5α protein (TRIM5α_rh_) in human cells renders them highly restrictive to infection by HIV-1 [3]. Unlike Fv1, TRIM5α acts at a stage prior to completion of reverse transcription [3],[20],[23],[24]. The human genome encodes a TRIM5α protein (TRIM5α_hu_) that restricts multiple retroviruses including N-tropic MLV (N-MLV), feline immunodeficiency virus (FIV), and equine infectious anemia virus (EIAV) but does not efficiently restrict HIV-1 [29], [30], [32]–[37]. TRIM5α cDNAs have now been cloned from multiple primate species; these differentially restrict infection by HIV-1, HIV-2, and SIV [28],[31].

Shortly after the identification of TRIM5α, a second HIV-1 restriction factor was identified in owl monkeys [4],[5]. This protein, TRIMCyp, is the apparent result of a LINE1-mediated retrotransposition event in which the cyclophilin A (CypA) mRNA was inserted into the TRIM5 locus resulting in a functional fusion protein [4]. TRIMCyp potently inhibits HIV-1 infection by interacting with an exposed loop on the surface of the CA via the CypA domain. The discovery of TRIMCyp provided a simple explanation for the ability of cyclosporin A (CsA), which inhibits CypA binding to CA, to render owl monkey cells permissive to HIV-1 infection [38]. Mutations in the CypA binding loop that result in a failure to bind CypA also result in a loss of restriction by TRIMCyp [4],[5]. More recently, novel TRIM5-CypA proteins have also been identified in other primate species [39]–[42].

TRIM5α and TRIMCyp are members of the tripartite motif family of proteins, which encode RING, B-Box, and coiled-coil (RBCC) domains [43]. TRIM5α is the longest of the three isoforms (α, γ, and δ) generated from the TRIM5 locus by alternative splicing of the primary transcript. While all three TRIM5 isoforms contain identical RBCC domains, the α-transcript also encodes the B30.2/SPRY domain required for recognition of the incoming viral capsid and restriction specificity [29], [30], [33], [34], [36], [44]–[46]. The coiled-coil domain promotes the multimerization of TRIM5α molecules that is required for efficient restriction [44],[47],[48]. While the precise function of the B-Box domain is unclear, deletion of this region results in total loss of restriction potential thus indicating its importance [44],[49]. The RING domain of TRIM5α is also required for full restriction activity, as mutants that lack this domain or in which proper folding is impaired are severely impaired for restriction and have altered cellular localization [3],[44],[49]. Substitution of RING domains from other human TRIM proteins results in changes in both the timing of restriction (i.e. pre- vs. post-reverse transcription) and the intracellular localization of the restriction factor [37], [50]–[52].

RING domains are commonly associated with ubiquitin ligase (E3) activity facilitating specific transfer of ubiquitin from various ubiquitin-conjugating (E2) proteins to substrates (reviewed in [53],[54]). Polyubiquitylation of proteins commonly targets them for intracellular degradation by proteasomes. TRIM5α can be ubiquitylated in cells [55], but a role for this modification in TRIM5α stability or restriction has not been established. The δ isoform of TRIM5, which encodes an identical RING domain to TRIM5α, exhibits E3 activity *in vitro* and mutation of the RING domain abolishes this activity [56]. The presence of a RING domain on TRIM5α suggested that the restriction factor might function by transferring ubiquitin to a core-associated viral protein, thus targeting it for proteasomal degradation. However, such a modification has not been detected, and the magnitude of restriction imposed by TRIM5α was not altered in cells in which the ubiquitination pathway was disrupted [57]. Nonetheless, recent studies have shown that proteasome inhibitors relieve the TRIM5α-dependent inhibition of reverse transcription, yet a block to HIV-1 nuclear entry remains [58],[59].

Based on these findings implicating the proteasome in TRIM5α-dependent retroviral restriction, we hypothesized that restriction by TRIM5α leads to proteasomal degradation of a TRIM5α-viral protein complex. Here we show that inoculation of TRIM5α-expressing cells with a restricted retrovirus results in accelerated degradation of TRIM5α itself. Destabilization of TRIM5α was tightly correlated with the ability of the restriction factor to block infection by the incoming virus. Proteasome inhibitors prevented HIV-1-induced degradation of TRIM5α_rh_ when added to cells prior to virus inoculation. These data suggest a functional link between proteasomal degradation of TRIM5α and the ability of TRIM5α to restrict an incoming retrovirus.

## Results

### Exposure of Cells to HIV-1 Destabilizes TRIM5α

**Figure 1:**
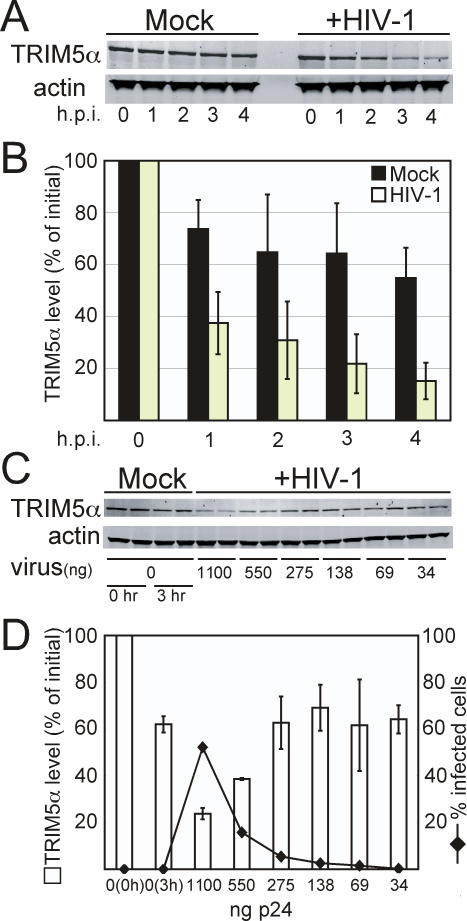
Destabilization of TRIM5α upon challenge of cells with HIV-1. (A) Immunoblot analysis of TRIM5α in cells challenged with HIV-1. 293T cells expressing HA-tagged TRIM5α_rh_ were pretreated for 1 hour with cycloheximide at 37°C. Cells were then challenged with stock solutions of HIV-1(VSV) or media alone (Mock). (B) Quantitation of TRIM5α levels utilizing Odyssey Band imaging software. The TRIM5α levels are expressed as a percentage of the ratio of TRIM5α:actin signal to the zero hour sample TRIM5α:actin signal. Shown are the mean values and standard deviations obtained in four independent experiments. h.p.i. = Hours post infection. (C) Immunoblot analysis of TRIM5α in cells challenged with HIV-1. 293T cells expressing HA-tagged TRIM5α_rh_ were pretreated for 1 hour with cycloheximide at 37°C. Cells were then challenged with media alone (Mock) or the indicated amount of pseudotyped HIV-GFP expressed as quantity of p24 (CA) for a period of three hours. (D) Relationship of TRIM5α level to permissivity of target cells. A portion of the cultures harvested in (C) were recultured for 48 hours and subsequently analyzed for GFP expression by flow cytometry. Shown are the mean values for the two replicates for both TRIM5α levels and extent of infection.

We hypothesized that TRIM5α itself might be degraded as a consequence of the post-entry restriction process. To test this, TRIM5α_rh_-expressing 293T cells were cultured in the presence of cycloheximide to arrest protein synthesis and then challenged with VSV-G-pseudotyped HIV-1 particles. At various times post-infection, cells were harvested for analysis of TRIM5α levels by quantitative immunoblotting. In control cells not exposed to virus, the TRIM5α level declined at a slow rate, eventually leveling off to 55% of the original level after 4 hours (Figure 1A). By contrast, inoculation with HIV-1 induced a more rapid decrease in the TRIM5α level resulting in 85% loss after 4 hours. Analysis of data from 4 experiments indicated that the decay of TRIM5α was significantly faster in the HIV-1-inoculated cultures relative to the control (Figure 1B). The stability of TRIM5α in our cells differs in terms of time as compared to previously published reports using Hela cells [55]. In additional studies we observed a similar destabilizing effect of HIV-1 exposure on TRIM5α_rh_ in HeLa cells (data not shown).

Exposure of target cells to saturating levels of virus or VLPs can overcome restriction by TRIM5α. To determine whether the decay of TRIM5α_rh_ was related to saturation of restriction, we inoculated TRIM5α_rh_–expressing cells with various doses of a GFP-encoding virus in the presence of cycloheximide for a fixed period of time and harvested the cells to quantify TRIM5α levels. To probe the relationship between saturation of restriction and TRIM5α degradation, a portion of the harvested cells were replated and cultured for 48 hours, and the extent of infection determined by flow cytometric analysis of GFP expression. The results showed that the ability to detect degradation of TRIM5α_rh_ was strongly dependent on the dose of virus used (Figure 1C). Furthermore, the TRIM5α level following inoculation was inversely related to the overall extent of infection (Figure 1D). These results indicate that HIV-1-induced degradation of TRIM5α is correlated with saturation of restriction, likely due to a requirement to engage most of the restriction factor to detect the loss of the protein.

### Human TRIM5α Stability is Not Affected by HIV-1

**Figure 2:**
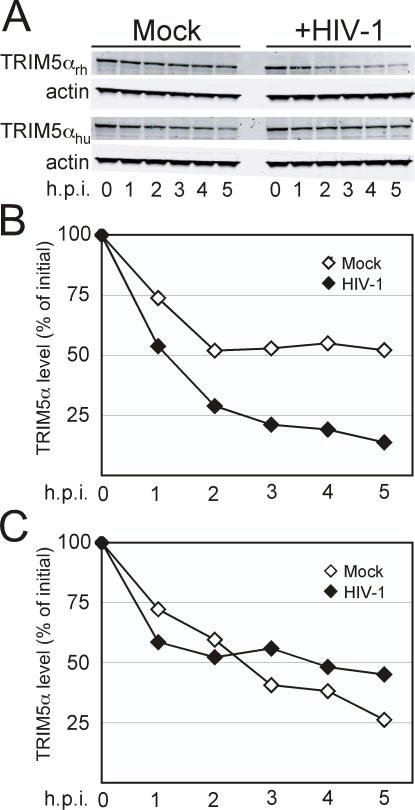
Rhesus macaque, but not human TRIM5α, is less stable in cells challenged with HIV-1. (A) 293T cells expressing HA-tagged TRIM5α_rh_ or TRIM5α_hu_ were pretreated for 1 hour with cycloheximide then exposed to VSV-G pseudotyped HIV-1 or media alone as in Figure 1. TRIM5α was detected by immunoblotting with HA-specific antibody. Integrated intensity values for the individual bands are shown in Figure S1. (B and C) Quantitation of TRIM5α_rh_ (B) and TRIM5α_hu_ levels utilizing Odyssey imaging software. Data shown are from one representative of three independent experiments.

Human TRIM5α does not efficiently restrict HIV-1 infection. To further probe the link between restriction and TRIM5α destabilization, we analyzed the stability of the human TRIM5α protein following challenge of cells with HIV-1. As previously shown in Figure 1, HIV-1 challenge of TRIM5α_rh_-expressing 293T cells resulted in a more rapid loss of the protein vs. mock-infected cells (Figure 2A and B). TRIM5α_hu_ was intrinsically less stable than TRIM5α_rh_, as indicated by its more rapid decay in the mock-infected cultures (Figure 2B and C). However, inoculation with HIV-1 did not result in further destabilization of TRIM5α_hu_, indicating that the HIV-1-induced degradation of TRIM5α_rh_ is not a nonspecific cellular response to the viral challenge. These results suggest that the loss of TRIM5α_rh_ depends on its ability to recognize the HIV-1 core.

### Exposure to Restriction-Sensitive HIV-1 Destabilizes TRIMCyp

**Figure 3:**
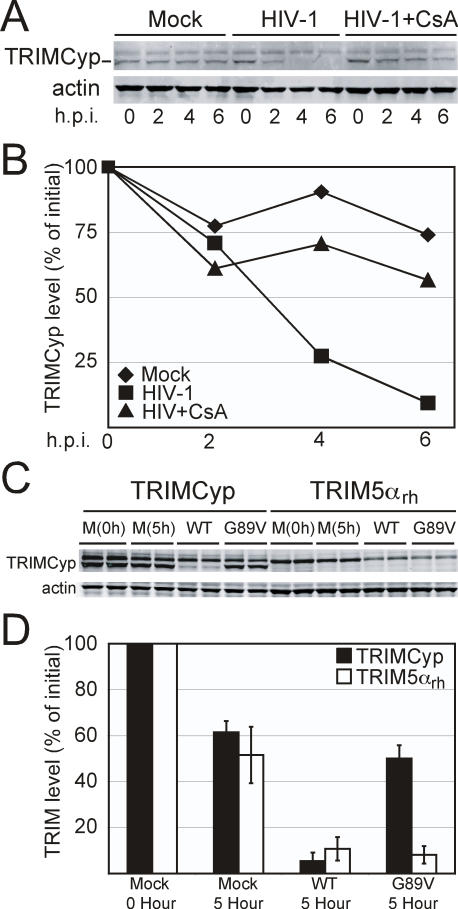
TRIMCyp is destabilized in cells challenged with HIV-1. (A) 293T cells expressing the myc-His_6_ tagged TRIMCyp were pretreated for 1 hour with cycloheximide. Cells were then challenged with HIV-1(VSV) and either ethanol carrier (HIV-1) or CsA (HIV-1+CsA), or with media alone (Mock). All stocks contained cycloheximide. (B) Quantitation of TRIMCyp levels as described in the legend to Figure 1B. Data in this figure are from one of two independent experiments. (C) 293T cells expressing either myc-His_6_ tagged TRIMCyp or HA-tagged TRIM5α_rh_ were pretreated for 1 hour with cycloheximide. Cells were then challenged for 5 hours with stock solutions of VSV-G pseudotyped HIV-GFP (WT), VSV-G pseudotyped HIV-GFP with the G89V capsid mutation (G89V), or medium alone (M). (D) Quantitation of TRIMCyp and TRIM5α levels as described in legend to Figure 1B. Data in (D) are expressed as the mean values of four determinations from two experiments, with error bars representing one standard deviation.

The owl monkey restriction factor TRIMCyp restricts HIV-1 by binding to an exposed loop on the surface of CA. Restriction can be prevented by addition of CsA or amino acid substitutions in CA that reduce CypA binding. We therefore asked whether TRIMCyp would also be destabilized following encounter of HIV-1. 293T cells expressing TRIMCyp were treated with cycloheximide and then challenged with VSV-G pseudotyped HIV-1 particles. As a control, parallel cultures were inoculated in the presence of a CsA concentration known to abolish TRIMCyp restriction of HIV-1. In the control mock-inoculated cells, TRIMCyp was stable in the cells during the six-hour time course (Figure 3A). Challenge with HIV-1 resulted in accelerated loss of TRIMCyp. In the cultures containing CsA, the HIV-1-induced loss of TRIMCyp was markedly reduced (Figure 3B).

Next we asked whether the HIV-1-induced degradation of TRIMCyp is correlated with the specificity of restriction. HIV-1 containing the G89V mutation in the CypA binding loop of CA is incapable of binding CypA and is also not restricted by TRIMCyp. However, this viral mutant is susceptible to TRIM5α_rh_ restriction. Parallel cultures of 293T cells expressing either TRIMCyp or TRIM5α_rh_ were treated with cycloheximide and then challenged with equivalent quantities of VSV-G pseudotyped HIV-GFP particles or the G89V CA mutant virus. As seen in Figure 3C, exposure to wild type HIV-1 induced accelerated loss of both TRIMCyp and TRIM5α_rh_. By contrast, exposure to the G89V mutant particles resulted in loss of TRIM5α_rh_ but not TRIMCyp. These results indicate that exposure of cells to HIV-1 results in destabilization of TRIMCyp by a mechanism requiring recognition of the incoming HIV-1 core by the restriction factor.

### Human TRIM5α is Destabilized Upon Encounter of N-tropic MLV

**Figure 4:**
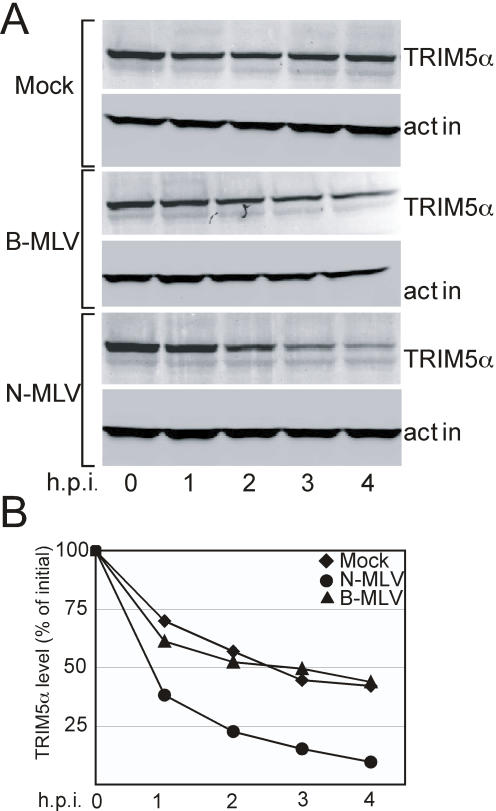
Human TRIM5α is destabilized upon challenge of cells with N-tropic but not B-tropic MLV. (A) 293T cells expressing HA-tagged TRIM5α_hu_ were pretreated for 1 hr with cycloheximide and exposed to stocks of VSV-G-pseudotyped N- or B-tropic MLV or media alone (Mock). Integrated intensity values for the individual bands are shown in Figure S3. (B) Quantitation of TRIM5α levels utilizing Odyssey Band imaging software. Data shown are from one representative of three independent experiments.

TRIM5α_hu_ cannot restrict HIV-1 or B-tropic MLV but potently restricts N-MLV. To further test the link between TRIM5α destabilization and retrovirus restriction, we challenged 293T cells stably expressing TRIM5α_hu_ with N- and B-tropic MLV viruses and measured TRIM5α levels following infection. The GFP-transducing N- and B-tropic MLV stocks were first titrated on nonrestrictive CrFK cells (Figure S2, detailed in Text S1) then normalized to ensure equivalent dosing. Mock-treated cells lost TRIM5α_hu_ at a slow rate (t_1/2_∼2.5 h; Figure 4A). Challenge with B-MLV did not significantly affect the rate of TRIM5α_hu_ decay (Figure 4A). By contrast, cells challenged with an equivalent quantity of N-MLV showed accelerated loss of TRIM5α_hu_ (t_1/2_<1 h) (Figure 4A and 4B). The relative band intensities of the TRIM5α levels for this experiment were calculated and are represented in the graph in Figure 4B. These results, together with the TRIM5α and TRIMCyp data, establish a strong correlation between virus-induced TRIM5α destabilization and the specificity of restriction.

### Virus-induced TRIM5α Destabilization is Correlated with Lentiviral Restriction in Old and New World Monkeys

**Figure 5:**
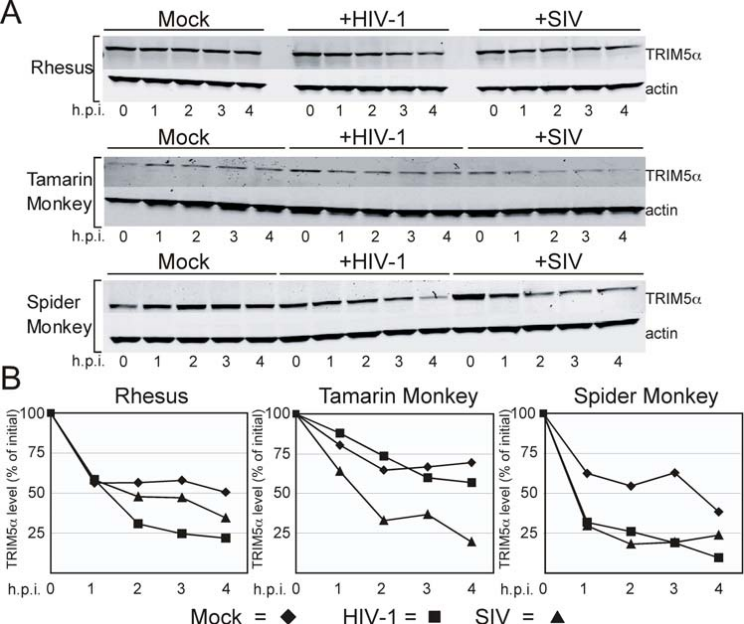
Destabilization of primate TRIM5α proteins is correlated with restriction of HIV-1 and SIVmac239. (A) 293T cells expressing HA-tagged TRIM5α_rh_, TRIM5α_tam_ (tamarin monkey), TRIM5α_sp_ (spider monkey), and were pretreated for 1 hour with cycloheximide then exposed to stocks of VSV-G pseudotyped HIV-GFP, SIV-GFP, or media alone (Mock). (B). Quantitation of relative TRIM5α levels. Data shown are from one representative of three independent experiments.

TRIM5α proteins from different primates differ in their ability to restrict specific lentiviruses. For example, tamarin monkey TRIM5α (TRIM5α_tam_) restricts SIV_mac_ but not HIV-1, while spider monkey TRIM5α (TRIM5α_sp_) restricts both viruses. To further test the correlation between virus-induced loss of TRIM5α and antiviral specificity, we stably expressed the TRIM5α_tam_ and TRIM5α_sp_ proteins in 293T cells and challenged them with equivalent titers of VSV-pseudotyped HIV-1 and SIV_mac239_ GFP reporter viruses (as determined by titration on permissive CrFK cells). The cell lines were found to restrict the respective viruses by at least ten-fold (data not shown). Immunoblot analysis of post-nuclear lysates revealed that TRIM5α_rh_ was specifically destabilized when challenged with HIV-1 but not upon SIV_mac_ challenge (Figure 5A). By contrast, the SIV-restrictive TRIM5α_tam_ was destabilized only in response to SIV_mac_ challenge (Figure 5A). TRIM5α_sp_, which restricts both viruses, was degraded in response to challenge with either virus (Figure 5A and B). These results further strengthen the correlation between the specificity of retrovirus restriction and virus-induced destabilization of TRIM5α.

### HIV-1-Induced Destabilization of TRIM5α Requires Proteasome Activity

**Figure 6:**
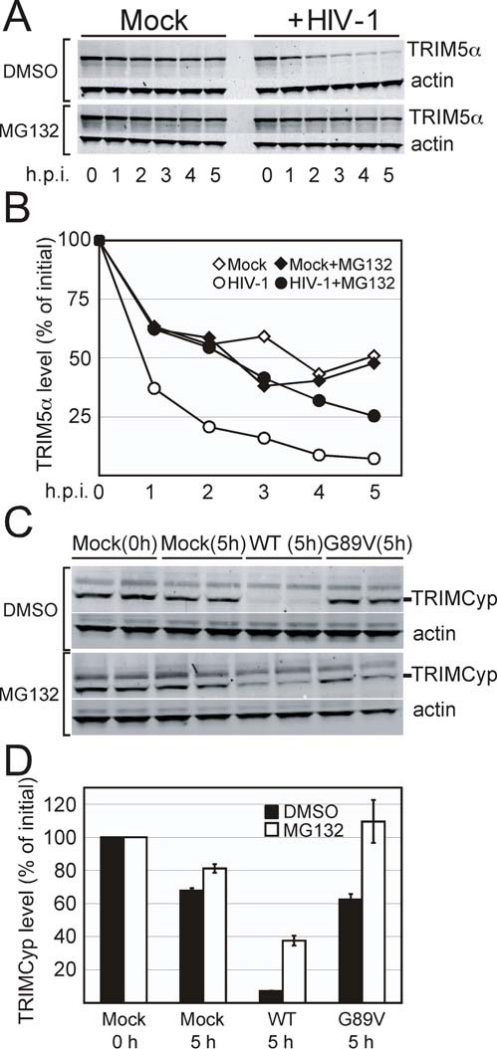
HIV-1-induced destabilization of TRIM5α is blocked by the proteasome inhibitor MG132. (A and B) 293T cells expressing HA-tagged TRIM5α_rh_ were cultured for 1 hour with cycloheximide and DMSO (Mock) or MG132 then exposed to HIV-1(VSV) with DMSO or MG132 (open or filled circles, respectively) or D10 media containing DMSO or MG132 (open or filled diamonds, respectively). Panel B shows quantitation of TRIM5α levels utilizing Odyssey imaging software. Data in this figure are from one of two independent experiments. (C). 293T cells expressing myc-His_6_-tagged TRIMCyp were cultured for 1 hour with cycloheximide and DMSO or MG132. The cells were then exposed for 5 hours to; VSV-G-pseudotyped HIV-GFP with either DMSO or MG132, VSV-G-pseudotyped HIV-GFP with the G89V capsid mutation (G89V) with either DMSO or MG132, or media alone containing DMSO or MG132 (Mock). (B) Quantitation of TRIMCyp levels utilizing Odyssey imaging software. Data in this figure are expressed as the average of the duplicate determinations, with the error bars depicting the range of values.

A major mechanism for cellular protein degradation is via the 26S proteasome. Previous studies have shown that the turnover of TRIM5α is dependent on cellular proteasome activity. Furthermore, inhibition of proteasome activity overcomes the early block to reverse transcription imposed by TRIM5α. We asked whether HIV-1-induced destabilization of TRIM5α_rh_ is dependent on proteasome activity. As previously reported [55], treatment of cells with the proteasome inhibitor MG132 resulted in an accumulation of TRIM5α protein (Figure 1, 0 H.p.i.). MG132 also prevented the HIV-1-induced destabilization of TRIM5α_rh_ (Figure 6A and B). Additional studies revealed that epoxomicin, a more specific proteasome inhibitor, also blocked the HIV-1-induced degradation of TRIM5α_rh_ (data not shown). By contrast, infection by HIV-1 in the presence of the S-cathepsin inhibitor E64 did not prevent HIV-1-induced TRIM5α_rh_ degradation (data not shown), suggesting that endosomal proteases are not responsible for TRIM5α_rh_ destabilization. We conclude that the virus-induced degradation of TRIM5α is dependent on cellular proteasome activity.

To determine whether HIV-1-induced destabilization of TRIMCyp depends on proteasome activity, we challenged TRIMCyp-expressing 293T cells with either restricted HIV-GFP or unrestricted HIV.G89V-GFP in the presence or absence of MG132. As shown in Figure 6C, MG132 prevented the HIV-1-induced loss of TRIMCyp. Infection with the unrestricted G89V virus did not alter TRIMCyp stability, while addition of MG132 stabilized the restriction factor.

### HIV-1-Induced Destabilization of Endogenous TRIM5α in Primate Cells

**Figure 7:**
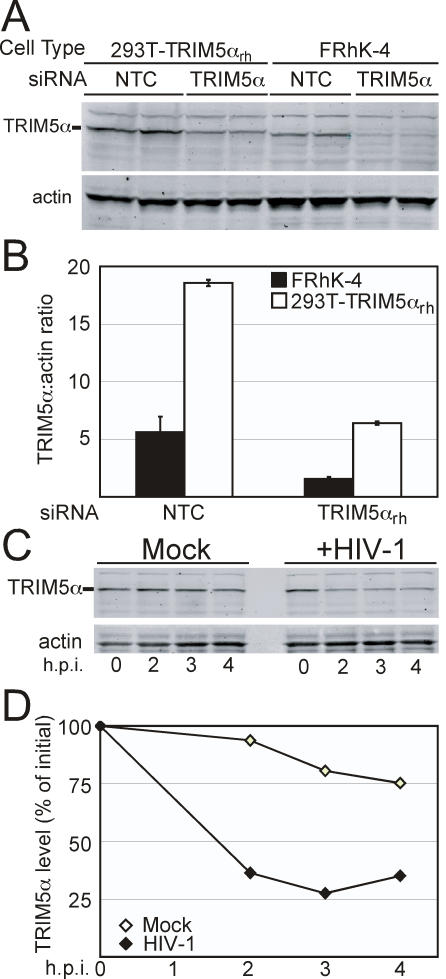
HIV-1 specifically induces destabilization of TRIM5α in rhesus macaque cells. (A) Duplicate cultures of 293T-TRIM5α_rh_ and FRHK-4 cells were transfected on two consecutive days with siRNAs specific for TRIM5α_rh_ (TRIM5α) or a non-targeting control siRNA (NTC). 72 hours after the second transfection, proteins were extracted and analyzed by immunoblotting with a TRIM5α–specific monoclonal antibody. (B) Quantitation of TRIM5α_rh_ levels in siRNA-transfected cells by Odyssey imaging software. TRIM5α_rh_ levels following siRNA knockdown are expressed as the average of the duplicate determinations with the error bars depicting the range of values. (C) FRhK-4 cells were challenged with HIV-GFP(VSV) or media alone (Mock). Zero hour timepoint represents TRIM5α levels in uninfected cells. (D) Quantitation of TRIM5α_rh_ levels. Results shown are from one representative of three independent experiments.

All of the previous experiments studying TRIM5α stability were conducted in transduced 293T cell lines in which TRIM5α was detected by virtue of a hemagglutinin epitope tag. In this setting, it was necessary to treat the cells with cycloheximide to detect virus-induced degradation of the restriction factor, potentially leading to artifacts due to general inhibition of protein synthesis. Virus titration experiments demonstrated markedly greater restriction in the transduced cells vs. rhesus macaque FRhK-4 cell line, indicating that the 293T cells overexpress TRIM5α_rh_ (our unpublished observations). Furthermore, while cycloheximide treatment had only a minor effect on restriction in FRhK-4 cells, the drug markedly reduced restriction in 293T cells (Figure S4). To probe the physiological relevance of our observations made in 293T cells, we sought a means of detecting endogenous TRIM5α protein in rhesus macaque cells. Using a monoclonal antibody against native TRIM5α for immunoblotting, we detected a band that was consistent in terms of molecular weight with TRIM5α_rh_ that was also absent in cells lacking TRIM5α_rh_ (data not shown). To confirm that the band is TRIM5α, we transfected FRhK-4 cells with either a TRIM5α_rh_ **-**specific siRNA duplex or a non-targeting control siRNA duplex and quantified the intensity of this band by immunoblotting. As shown in Figure 7A and B, transfection with TRIM5α_rh_ **-**specific siRNA resulted in a 72% decrease in intensity of the relevant band vs. FRhK-4 cells treated with the non-targeting control. Cells treated with the TRIM5α_rh_ **-**specific also exhibited a tenfold increase in permissiveness to infection with HIV-1 (data not shown). HIV-1 infection of FRhK-4 cells was not altered by treatment with the non-targeting siRNA control. As expected, treatment with either siRNA duplex did not affect permissiveness to SIV infection (data not shown). These results indicated that the monoclonal antibody is capable of detecting endogenous TRIM5α_rh_ in FRhK-4 cells. They further demonstrated that the transduced 293T cells express a 3.3 fold higher level of TRIM5α than FRhK-4 cells (Figure 7B).

We next sought to determine if endogenous TRIM5α_rh_ was destabilized by HIV-1 in rhesus macaque cells. FRhK-4 cultures were inoculated with HIV-1 in the presence or absence of cycloheximide and the stability of TRIM5α_rh_ in response to infection was analyzed by immunoblotting. Initial experiments showed no effect of cycloheximide treatment on TRIM5α_rh_ levels in HIV-1-exposed cells (data not shown); therefore the drug was removed in all subsequent experiments. We observed that TRIM5α_rh_ levels were stable in FRhK-4 cells over the 4 hour period (Figure 7C and D). Infection with HIV-1 resulted in accelerated decay of endogenous TRIM5α_rh_ in rhesus macaque cells without any requirement of inhibition of protein synthesis.

**Figure 8:**
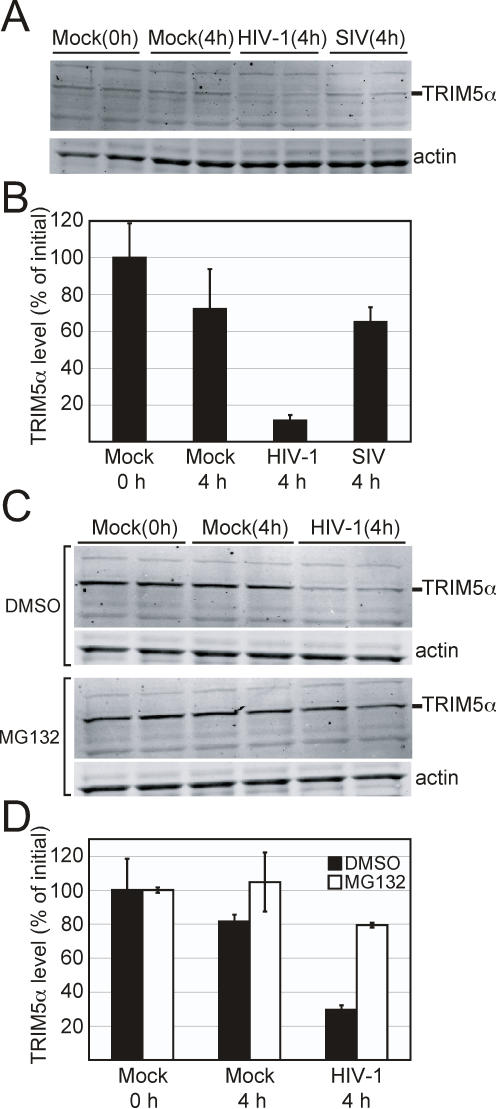
HIV-1-induced turnover of TRIM5α in primate cells specific to restriction and is blocked by the proteasome inhibitor MG132. (A) Duplicate cultures of FRhK-4 cells were exposed to stocks of VSV-G-pseudotyped HIV-GFP (HIV), SIV-GFP (SIV), or media alone (Mock) for a period of 4 hours then harvested and TRIM5α_rh_ levels quantified by immunoblotting. The zero hour sample corresponds to uninfected cells. (B) Quantitation of TRIM5α_rh_ levels in (A) utilizing Odyssey imaging software. (C) Duplicate cultures of FRhK-4 cells were treated for 1 hour with DMSO or MG132, then exposed for 4 hours to: VSV-G-pseudotyped HIV-GFP with either DMSO or MG132, or media alone containing DMSO or MG132 (Mock). (D) Quantitation of TRIM5α_rh_ levels utilizing Odyssey imaging software. Data in this figure are expressed as the averages of the two determinations, with the error bars spanning the range of values.

We next sought to determine if the loss of TRIM5α_rh_ was specifically due to restriction or was a non-specific effect resulting from viral infection. In the absence of cycloheximide we infected FRhK-4 cells with equivalent titers of HIV-1 or SIVmac239 GFP reporter viruses. As seen in Figure 8A and B, infection with HIV-1 resulted in a potent loss of TRIM5α_rh_ while infection with SIV resulted in only a slight loss of TRIM5α_rh_ as compared to the control cells. We conclude that infection by HIV-1 results in a rapid loss of TRIM5α_rh_ in target cells and that this loss is directly related to the ability of TRIM5α_rh_ to restrict infection by the incoming virus.

### HIV-1-Induced Destabilization of Endogenous TRIM5α Requires Active Proteasomes

We sought to determine if inhibition of proteasome function would restore TRIM5α_rh_ stability in rhesus macaque cells. FRhK-4 cells were exposed to HIV-1 in the presence or absence of MG132 for a period of four hours, and the levels of TRIM5α_rh_ were measured by immunoblotting. As can be seen in Figure 8C and D, MG132 stabilized TRIM5α_rh_ in HIV-1-exposed cells. Flow cytometry analysis of GFP signal in a small subset of the infected cells showed no difference in infection levels resulting from inhibition of proteasome function, which is consistent with previously published results. These results indicate that HIV-1-induced destabilization of TRIM5α_rh_ in rhesus macaque cells requires proteasome activity. They further suggest that the results we observed with TRIM5α-transduced 293T cells are unlikely to be an artifact of cycloheximide treatment.

## Discussion

While it is well established that TRIM5α limits the host range of many retroviruses, the precise mechanism of restriction remains undefined. TRIM5α can specifically associate with assemblies of HIV-1 CA-NC protein *in vitro*, and genetic evidence indicates that TRIM5α and TRIMCyp require an intact or semiintact viral capsid for binding [60],[61]. However, the detailed molecular consequences of the binding interaction to the viral core remain poorly defined. Two lines of evidence have implicated the ubiquitin-proteasome system in restriction. First, the δ isoform of TRIM5, which has a RING domain identical to that of TRIM5α, exhibits E3 activity *in vitro* [56]. Deletion or mutation of the RING domain in TRIM5α results in significant loss of restriction efficacy [44],[49]. TRIM5α is ubiquitinated in cells, although a role of this modification in retrovirus restriction has not been established [55]. Second, inhibition of proteasome activity alters the stage at which TRIM5α-mediated restriction occurs [58],[59]. The latter observation led us to hypothesize that the proteasome may participate in restriction by degrading a complex of TRIM5α with one or more incoming viral proteins. To test this, we asked whether exposure of cells to HIV-1 alters the stability of TRIM5α_rh_. We observed that inoculation with HIV-1 results in an accelerated turnover of the restriction factor. Similar effects were observed in both 293T and HeLa cells (data not shown), suggesting that TRIM5α destabilization is not specific to a unique cell type. HIV-1 challenge resulted in destabilization of TRIM5α_rh_ but not TRIM5α_hu_. Likewise, TRIM5α_hu_ was destabilized by inoculation of cells with restriction-sensitive N-MLV particles but not by unrestricted B-MLV. Similar results were seen in cells expressing the HIV-1-specific restriction factor TRIMCyp. Treatment of target cells with CsA, which blocks TRIMCyp restriction of HIV-1, or infection with virus containing mutations that prevent CypA binding [4],[5],[38], did not affect TRIMCyp stability. Specific loss of TRIM5α from cells expressing different primate alleles of the protein also correlated very well with the ability of those alleles to restrict HIV or SIV. The HIV-1-induced destabilization of TRIM5α_rh_ and TRIMCyp was prevented by inhibition of cellular proteasome activity. Destabilization of TRIM5α_rh_ by HIV-1 was also observed in a primate derived cell line without the need of cycloheximide to inhibit protein synthesis. This destabilization was specific for the restricted HIV-1 and was not observed in cells infected with an unrestricted virus. Inhibition of proteasome function restored TRIM5α_rh_ stability in response to infection by HIV-1 in the rhesus macaque cells. We conclude that TRIM5-related restriction factors are targeted for degradation by a proteasome-dependent mechanism following encounter of a restriction-sensitive retroviral core.

TRIM5α forms heterogenous structures in cells referred to as cytoplasmic bodies (CBs). While the role of CBs in restriction is unclear, TRIM5α protein in these structures rapidly exchanges with soluble TRIM5α, indicating that the protein is highly dynamic within cells [62]. We observed that most of the cellular TRIM5α can be degraded in response to exposure to a restriction-sensitive retrovirus, which implies that a majority of cellular TRIM5α molecules can engage incoming viral cores. If the CB-associated TRIM5α is inaccessible to incoming virus, our observation that a restricted virus can induce degradation of the majority of the TRIM5α molecules suggests that this protein rapidly redistributes to a compartment accessible to incoming virus.

TRIM5α and TRIMCyp are subject to proteasome-dependent turnover under steady-state conditions, yet its rapid turnover is not a prerequisite for restriction activity [55],[63]. Accordingly, proteasome inhibitors do not overcome restriction ([57]; Figure S5). Nonetheless, the effect of virus exposure on TRIM5α stability had heretofore not been reported. While alterations of specific individual portions of TRIM5α may alter its intrinsic stability, our results indicate that TRIM5α encounter with a restricted core results in degradation of the restriction factor by a proteasome-dependent mechanism.

Retrovirus uncoating is a poorly characterized process, but can be defined as the disassembly of the viral capsid following penetration of the viral core into the target cell cytoplasm. Studies of HIV-1 CA mutants indicate that the stability of the viral capsid is properly balanced for productive uncoating in target cells: mutants with unstable capsids are impaired for viral DNA synthesis, suggesting that premature uncoating is detrimental to reverse transcription [64]. Thus a plausible mechanism for restriction is that binding of TRIM5α to the viral capsid inhibits infection directly by physically triggering premature uncoating in target cells [65],[66]. In this model, TRIM5α, perhaps with one or more cofactors, promotes the physical decapsidation of the virus core independently of proteolysis. Consistent with this view are studies demonstrating that TRIM5α restriction is associated with decreased recovery of sedimentable CA protein in lysates of acutely-infected cells [65],[66]. However, these studies fell short of demonstrating that the sedimentable CA protein was associated with intact viral cores. Furthermore, a recent study reported that treatment of cells with proteasome inhibitors prevented TRIM5α-dependent loss of particulate CA protein [67], indicating the potential involvement of proteasome activity in TRIM5α-induced virus uncoating.

Other studies further implicate the activity of the proteasome in TRIM5α-dependent restriction. Inhibition of proteasome activity rescues HIV-1 reverse transcription in TRIM5α-expressing cells, revealing a downstream block to nuclear import mediated by the restriction factor [58],[59]. Engagement of the viral capsid by TRIM5α may lead to proteasomal degradation of a TRIM5α-CA complex, resulting in functional decapsidation of the viral core and a premature uncoating phenotype. Consistent with this model, TRIM5α restriction has been associated with decreased intracellular accumulation of HIV-1 CA [68]. In addition, a recent study of MLV particle-mediated RNA cellular transfer reported reduced accumulation of viral CA protein in cells in a manner that was correlated with restriction by TRIM5α, and this effect was reversed by proteasome inhibition [69]. Unfortunately, our own efforts to detect an effect of TRIM5α on the stability of the incoming HIV-1 CA have thus far yielded negative results; thus we are reluctant to conclude at this stage that a specific component of the viral core is degraded as a complex with TRIM5α. Another potential mechanism is that proteasomal engagement of TRIM5α bound to the virus core results in physical dissociation of CA from the core followed by its release from TRIM5α, thus leading to destruction of the restriction factor and decapsidation of the core but not necessarily degradation of CA [70]. Genetic evidence from abrogation-of-restriction studies indicates that TRIM5α binding requires an intact or semiintact viral capsid [60], suggesting that TRIM5α binding to CA is highly dependent on avidity resulting from multivalent interactions with the polymeric viral capsid. It is thus plausible that CA is released from TRIM5α following forced uncoating. This model is attractive in its ability to reconcile most, if not all, of the reported data regarding the mechanism of restriction by TRIM5α.

HIV-1 infection in many primate cell lines exhibits biphasic titration curves, and restriction can be abrogated *in trans* by high concentrations of VLPs, indicating that virus restriction is saturable. While it is generally assumed that the saturation occurs via sequestration of the restriction factor by the incoming virus, our results reveal another potential mechanism. Degradation of TRIM5α_rh_ by HIV-1 was tightly correlated with cellular susceptibility to infection by incoming virus, suggesting that loss of restriction at high virus input may occur via degradation of the restriction factor itself. Consistent with this view, treatment with MG132 resulted in a three-fold decrease in HIV-1 infection of FRhK-4 as well as OMK cells, while infection by unrestricted SIV was inhibited only marginally (Figure S5). This result, coupled with our observations of proteasome-dependent degradation of TRIM5α proteins in restrictive cells, suggests that depletion of TRIM5α via the proteasome contributes to the saturability of restriction.

The potential involvement of ubiquitylation in virus-induced degradation of TRIM5α degradation warrants further study. The autoubiquitylation of TRIM5δ observed *in vitro* suggests that TRIM5α may be ubiquitylated *in trans* upon polymerization of the restriction factor on a retroviral capsid. However, we have been unable to detect accumulation of cellular ubiquitylated TRIM5α species following HIV-1 inoculation either in the presence or absence of proteasome inhibitors (our unpublished observations). While many cellular proteins are regulated by ubiquitin-dependent proteolysis, ubiquitin-independent proteasomal degradation is also well documented (reviewed in [71]). Most E3 ligases are not degraded following ubiquitylation of a substrate, yet notable exceptions exist. The E3 enzyme Mdm2 is degraded following its ubiquitylation of its target, p53 [72], and the stability of several E3 ligases is related to their ubiquitylation status resulting from autoubiquitylation [73]–[75]. It will therefore be of interest to determine whether HIV-1-induced degradation of TRIM5α is dependent on host cell ubiquitylation and the TRIM5α RING domain.

The early post-entry stage of infection remains a fundamentally obscure part of the retrovirus life cycle. Our results provide novel evidence for a role for proteasome activity in TRIM5α restriction. Further mechanistic studies of TRIM5α may reveal novel approaches to antiviral therapy and fundamental insights into the molecular details of HIV-1 uncoating.

## Materials and Methods

### Plasmids

pLPCX-TRIM5α_rh_ (rhesus macaque), pLPCX-TRIM5α_hu_ (human), pLPCX-TRIM5α_sp_ (spider monkey), and pLPCX-TRIM5α_tam_ (tamarin monkey) were generous gifts from Dr. J. Sodroski [3],[31]. pCIG-N and pCIG-B were generous gifts from J. Stoye [76]. pNL4-3 was obtained from the NIH AIDS Research and Reference Reagent Program and the *env* gene inactivated as previously described [77]. pHIV-GFP [78], pSIV-GFP [23], and pCL-ampho [79] were gifts from D. Gabuzda, P. Bieniasz, and B. Naviaux, respectively. R9-G89V was made by PCR mutagenesis of the wild type HIV-1 provirus R9 utilizing site-specific primers and verified by sequencing. pHIV-G89V-GFP was made by transfer of the BssHII-EcoRI fragment of R9-G89V into the BssHII-EcoRI sites of pHIV-GFP and verified by restriction digest. pHCMV-G was provided by J. Burns [80]. pBABE-eGFP was created by transfer of the BamHI-EcoRI fragment from peGFP (Clontech) into the BamHI-EcoRI sites of pBABE-puro [81]. pBABE-rhTRIM5α and pBABE-huTRIM5α were generated by PCR amplification of the rhesus and human TRIM5α sequences from pLPCX-TRIM5α_rh_ and pLPCX-TRIM5α_hu_ using primers TRIM5α-1(S)-Eco 5′- GATCGAATTCAGCTACTATGGCTTCTGGAATCCTG-3′ and pTM1-TRIMHA-R 5′-GTCTCGAGTCAAGCGTAGTCTGGGACG-3′ (EcoRI and XhoI sites underlined). The PCR products were digested and ligated into the EcoRI and SalI sites in pBABE-puro. A TRIMCyp cDNA was generated from oligo dT-primed owl monkey kidney cell cDNA and PCR amplified using the TRIM5α-1(S)-Eco and primer 5′-CTAGCTCGAGTACAGAAGGAATGATCTGG-3′ (XhoI site underlined) specific to the 3′-UTR of the human cyclophilin A gene. This amplification results in a Arg to Gly substitution at codon 4 as compared to the original TRIMCyp cDNA. The product was ligated into the EcoRI-XhoI sites of plasmid CMX-PL1. The Myc-His_6_ tag was added to TRIMCyp by PCR amplification of CMX-PL1-TRIMCyp with TRIM5α-1(S)-Eco and primer 5′-GTCTCGAGAGAGCTTGGTGAGCACAGAGTCATGG-3′ (XhoI site underlined). This product was then digested with EcoRI and XhoI and ligated into pcDNA 3.1/myc-His A (Invitrogen). The TRIMCyp containing the myc-(His)_6_ epitope tag was then amplified from TRIMCyp-pcDNA3.1/myc-His A using TRIM5α-1(S)-Eco and the primer pcDNA3.1 HIS-Sal 5′-ACGTCGACTTTCAATGGTGATGGTGATGATGACC-3′, and the product digested with EcoRI and SalI and ligated into the corresponding sites in pBABE-puro. All constructs were verified via bidirectional DNA sequencing.

### Chemicals

MG132 and cycloheximide were purchased from Sigma-Aldrich and used at final concentrations of 25 µM and 50 µM, respectively. Cyclosporin A was purchased from CalBiochem used at 2.5 µM final concentration. Epoxomicin was purchased from Boston Biochem and used at 10 µM. The cathepsin inhibitor E64 was purchased from Sigma-Aldrich and was used at 40 µM.

### Cells and Viruses

FRhK-4 cells were purchased from the American Type Culture Collection. Cells were cultured in Dulbecco's modified Eagle's medium containing 10% fetal bovine serum and 1% penicillin/streptomycin. VSV-G-pseudotyped HIV-1_NL4.3_, HIV-GFP, and SIV-GFP viruses were produced by calcium phosphate transfection of 293T cells with proviral plasmid DNA (23 µg) and pHCMV-G (7 µg). N- and B-tropic MLV virus stocks were prepared by co-transfection of 23 µg pCIG-N or pCIG-B plasmids with pHCMV-G (7 µg) onto the cell line 293TeGFP. This cell line is a clone generated from 293T cells previously transduced with the retroviral vector pBABE-eGFP and isolated by limiting dilution and selected for high levels of GFP expression. Transfected cells were washed after 24 hours and replenished with fresh media. Supernatants were harvested 48–72 hours after transfection, clarified by passing through 0.45 µm filters, and stored in aliquots at −80°C. Retrovirus stocks for transduction of TRIM5α alleles were harvested from 293T cells transfected with the plasmids pCL-ampho (10 µg), the appropriate TRIM5α vector (15 µg), and pHCMV-G (5 µg). Viruses were collected 48 hours after transfection and used to transduce 293T cells. All 293T cell lines expressing TRIM5α proteins were polyclonal cell populations obtained by selection of transduced cells with puromycin. TRIMCyp-expressing cells were obtained by isolation of a single cell clone via limiting dilution. HIV-1 was strongly restricted in these cells, and restriction was prevented by the addition of 5 µg/ml cyclosporin A (CsA).

### Infection Protocol

Cells were seeded in 6-well plates at a density of 1 to 1.25×10^6^ cells/well and incubated overnight. Prior to infection, cultures were treated for 1 hour in 50 µM cycloheximide to block protein synthesis. In experiments involving proteasome inhibitors, cells were incubated with both cycloheximide and the appropriate inhibitor for 1 hour prior to infection. Viral stocks containing cycloheximide, polybrene (5 µg/mL), CsA (2.5 µM), and proteasome inhibitors were prewarmed to 37°C prior to addition to cells. After culturing for 1 hr, media from zero hour timepoints was removed and 1 ml of PBS was added. Cells were then detached from the plate by flushing, pelleted, washed in PBS, repelleted, and the pellets frozen at −80°C. Cells that were challenged with virus had media removed and replaced with viral stock and were returned to 37°C. Individual cultures were harvested hourly using same procedure as previously described for the zero hour timepoints. All cell pellets were frozen at −80°C prior to analysis. For experiments utilizing FRhK-4 cells the cells were seeded in 6 well plates at a density of 3×10^5^ cells/well and incubated overnight. Prewarmed viral stocks containing polybrene (5 µg/mL) were added the following day with a well harvested at the time of viral addition serving as the zero hour timepoint. Cells were incubated with the viral stock for the indicated time period then trypsinized, placed in fresh D10 media at a 1∶1 volume, pelleted, washed in 1 mL complete D10 media to inactivate trypsin, repelleted, washed 2 times in 1 mL PBS, then frozen at −80°C. In experiments with FRhK-4 cells involving MG132, the cells were incubated with inhibitor for one hour prior to viral addition with the zero hour timepoint being an uninfected well harvested after 1 hour pretreatment.

### siRNA Knockdown of TRIM5α_rh_

293T and FRhK-4 cells were seeded at a density of 2×10^5^ cells per well in 6-well plates and incubated overnight. 24 hours later, TRIM5α_rh_-specific siRNA [3], or a non-targeting control siRNA (Dharmacon), were diluted to a concentration of 3 µM in 1× siRNA buffer then transfected into cells using Dharmafect 1 transfection reagent and OptiMEM I (Gibco) according to manufacturers protocol (Dharmacon). Cells were then incubated overnight and retransfected with siRNAs again the following day utilizing the identical protocol. 48 hours after the first siRNA transfection the cells were removed from the 6-well plates and plated onto a 10 cm dish in complete D10 media at a ratio of 1 well to 1 10 cm dish and incubated for either 24 or 48 hours. 24 hours later, one 10 cm dish of either TRIM5α_rh_-specific siRNA treated cells or non-targeting control treated cells were trypsinized and replated in 24 well plates at a density of 2×10^5^ cells/well then incubated overnight. The following day the remaining two 10 cm dishes of siRNA treated cells were trypsinized, diluted 1∶1 in D10 media, pelleted, washed 1× in D10 media to inactivate trypsin, repelleted, washed 2× in 1 mL PBS per wash, repelleted, then frozen at −80°C. Cells that had been seeded the prior day in the 24 well plates were then infected with dilutions of HIV and SIV-GFP, incubated for 48 hours, then analyzed for GFP expression by flow cytometry.

### Protein Analyses

Cell pellets were thawed and lysed in a solution containing 100 mM Tris-HCl (pH 8.0), 100 mM NaCl, and 0.5% NP-40. Nuclei were pelleted via centrifugation at 16,000×*g* for 10 minutes and post-nuclear supernatants were removed. Protein levels were quantified via BCA assay (Pierce). Samples, normalized for total protein, were denatured in SDS and subjected to electrophoresis on 4–20% acrylamide gradient gels (BioRad). Proteins were transferred to nitrocellulose and probed with HA-epitope tag-specific rat monoclonal antibody (3F10, Roche) and Alexa Fluor 680 conjugated goat anti-rat IgG (Molecular Probes). Cells expressing TRIMCyp were probed with the myc epitope-specific mouse monoclonal antibody (9E10, Invitrogen) and Alexa Fluor 680-conjugated goat anti-mouse IgG (Molecular Probes). Proteins extracted from FRhK-4 cells were probed the TRIM5α-specific mouse polyclonal antibody (IMG-5354, Imgenex) and Alexa Fluor 680 conjugated goat anti-mouse IgG (Molecular Probes). All immunoblots were probed with β-actin-specific rabbit monoclonal antibody (A2228, Sigma) and IRDye800-conjugated goat anti-rabbit IgG (Rockland). Dilutions of antibodies were 1∶1000 and 1∶5000 for primary and secondary respectively with the exception of IMG-5354 which was used at a dilution of 1∶2000. Bands were detected by scanning blots with the LI-COR Odyssey Imaging System using both 700 and 800 channels, and integrated intensities were determined using the LI-COR Odyssey band quantitation software with the median top-bottom background subtraction method. The TRIM5α band intensities were then normalized to the signals from the corresponding β-actin bands. All signals were then expressed as a percentage of the initial TRIM5α/actin band intensity ratio.

### Genes used in this study

TRIM5α_rh_ (AY523632); TRIM5α_hu_ (AF220025); TRIMCyp (AY646198); TRIM5α_tam_ (AY740615); TRIM5α_sp_ (AY740616).

## Supporting Information

Figure S1 Integrated Intensity Values for Bands for immunoblot in Figure 2A.
(0.20 MB TIF)

Figure S2 Titration Curve of N- and B-Tropic MLV viruses on TRIM5α_hu_ and CrFK cells.
(0.05 MB TIF)

Figure S3 Integrated Intensity Values for Bands for immunoblot in Figure 4A.
(0.10 MB TIF)

Figure S4 Effects of cycloheximide on HIV-1 restriction in 293T-TRIM5α_rh_ and FRhK-4 cells.
(0.29 MB TIF)

Figure S5 Effects of MG132 on HIV-1 restriction in simian cell lines.
(0.46 MB EPS)

Text S1 Supporting Methods for Figures S2, S4, and S5.
(0.03 MB DOC)
