# Supplementary figures and images for: Proteasomal Degradation of TRIM5α during Retrovirus Restriction
Source: PLoS Pathog. 2008 May 23;4(5):e1000074. doi: 10.1371/journal.ppat.1000074 (PMC2374908; doi:10.1371/journal.ppat.1000074)

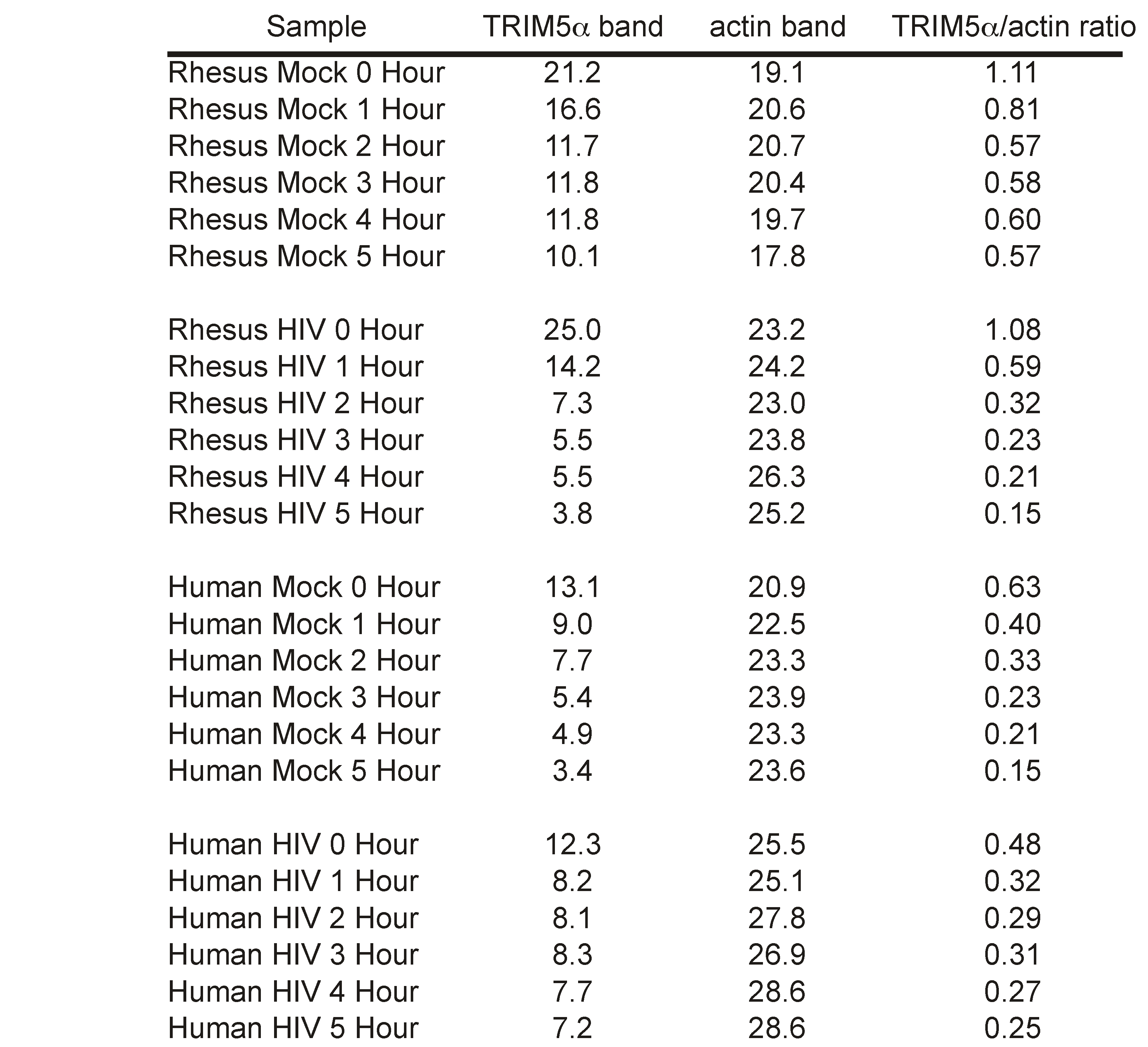

Supplement: Figure S1 — Integrated Intensity Values for Bands for immunoblot in Figure 2A. (0.20 MB TIF) [file ppat.1000074.s001.tif]

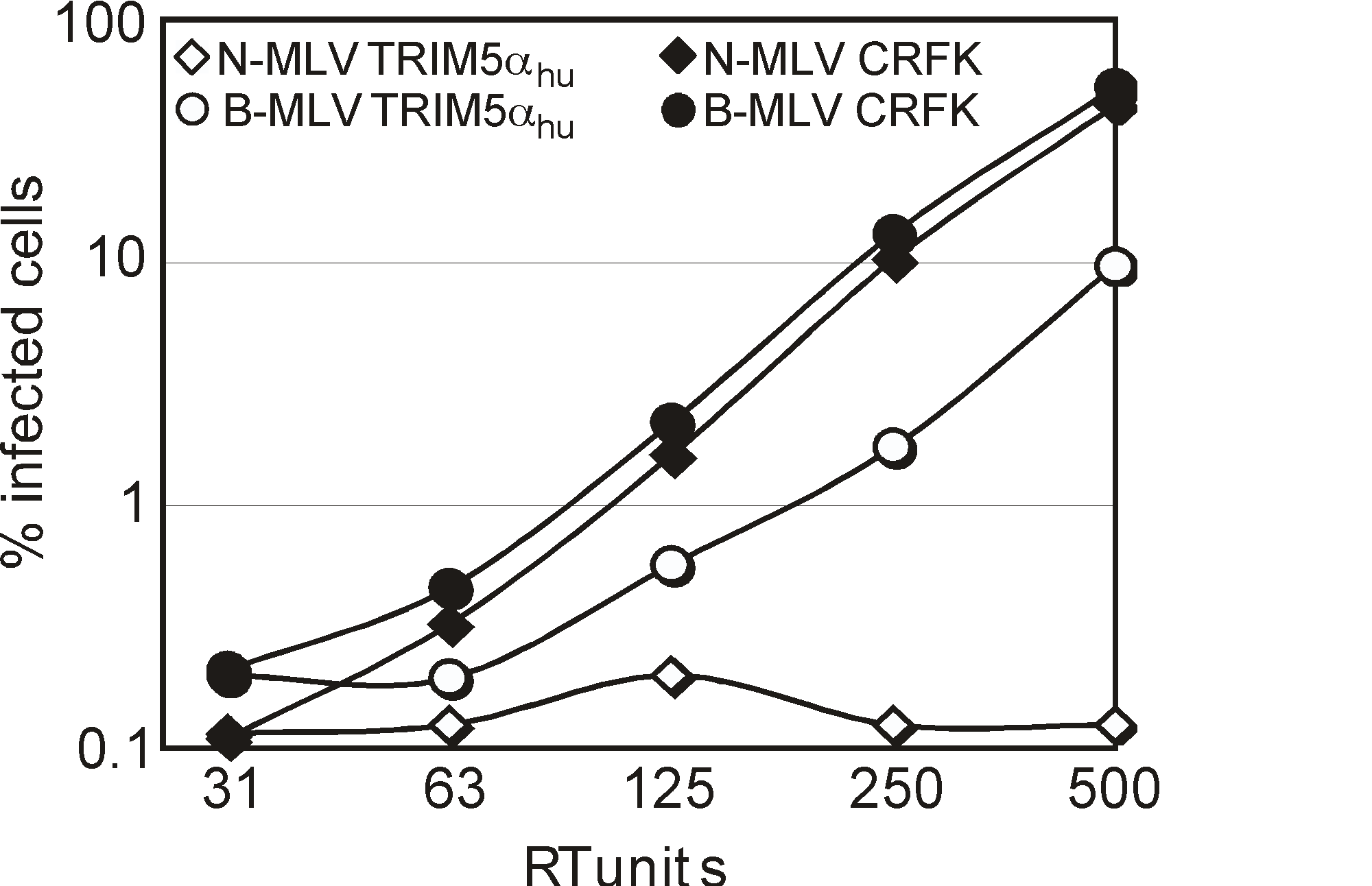

Supplement: Figure S2 — Titration Curve of N- and B-Tropic MLV viruses on TRIM5αhu and CrFK cells. (0.05 MB TIF) [file ppat.1000074.s002.tif]

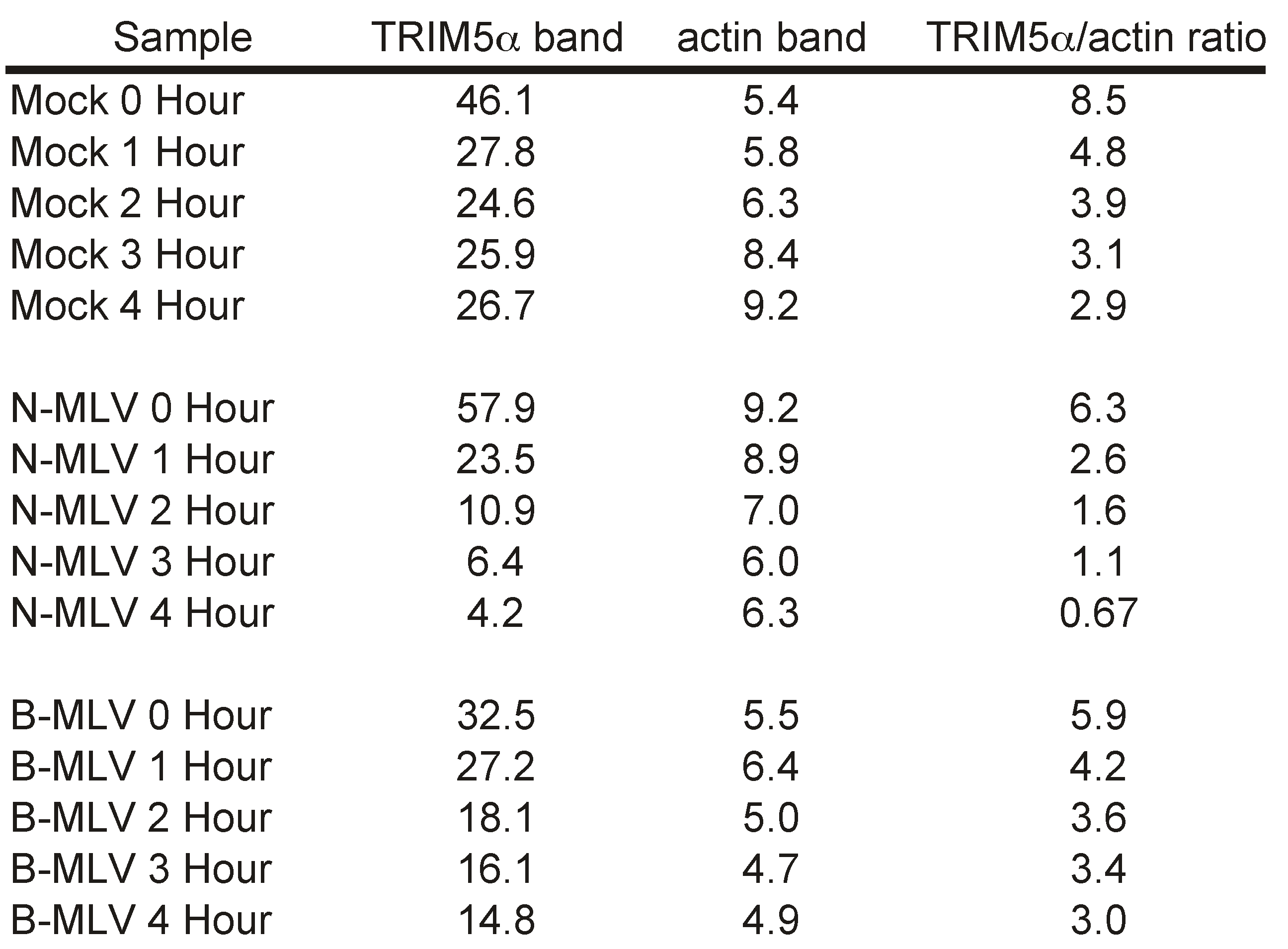

Supplement: Figure S3 — Integrated Intensity Values for Bands for immunoblot in Figure 4A. (0.10 MB TIF) [file ppat.1000074.s003.tif]

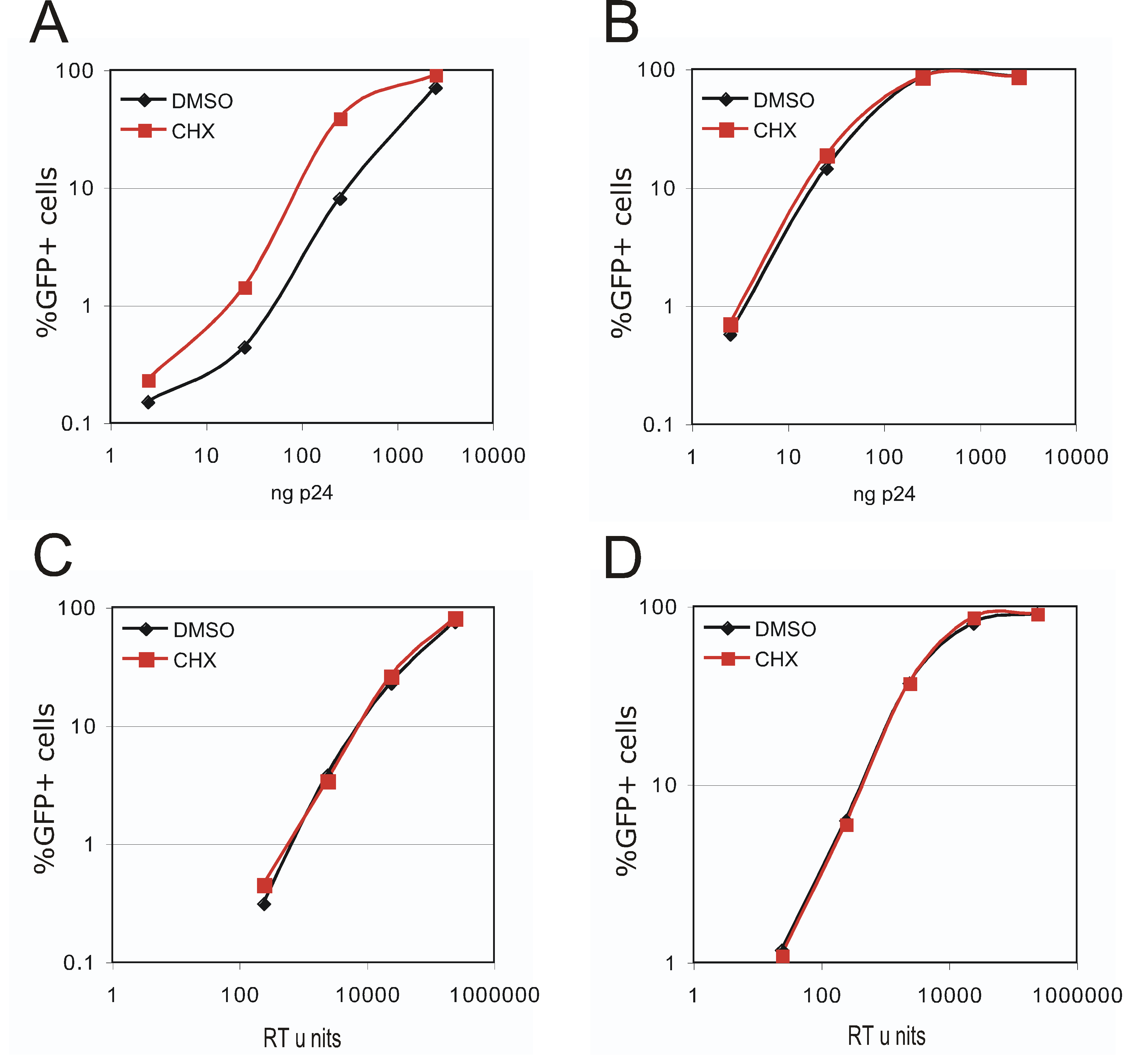

Supplement: Figure S4 — Effects of cycloheximide on HIV-1 restriction in 293T-TRIM5αrh and FRhK-4 cells. (0.29 MB TIF) [file ppat.1000074.s004.tif]
