# Supplementary material for: Proteasomal Degradation of TRIM5α during Retrovirus Restriction
Source: PLoS Pathog. 2008 May 23;4(5):e1000074. doi: 10.1371/journal.ppat.1000074 (PMC2374908; doi:10.1371/journal.ppat.1000074)
Supplement: Text S1 — Supporting Methods for Figures S2, S4, and S5. (0.03 MB DOC) [file ppat.1000074.s006.doc]

**Supporting Information.**

**Figure S1. Integrated Intensity Values for Bands for immunoblot in Figure 2A.**

**Figure S2. Titration Curve of N- and B-Tropic MLV viruses on TRIM5α_hu_ and CrFK cells.**

**Figure S3. Integrated Intensity Values for Bands for immunoblot in Figure 4A.**

**Figure S4. Effects of cycloheximide on HIV-1 restriction in 293T-TRIM5α_rh_ and FRhK-4 cells.**

**Figure S5. Effects of MG132 on HIV-1 restriction in simian cell lines.**

**Supporting Method for Figure S2**

CrFK and TRIM5α_hu_-expressing 293T cells were plated at 2x10^5^ cells per well in 24 well plates and incubated overnight. The following day, serial dilutions of N- and B-tropic MLV virus stocks were made in D10 media with polybrene (5 μg/mL) and added to each well of both cell types in duplicate. Cells were incubated for 24 hours after which fresh media (0.5 mL) was added to each well. 48 hours after viral infection the cells were trypsinized, fixed in 4% paraformaldehyde, and analyzed by FACS. Individual data points represent the average number of GFP^+^ cells from the two replicates for each viral dilution.

**Supporting Method for Figure S4**

293T- TRIM5α_rh_ (A and C) and FRhK-4 (B and D) cultures were pre-treated for one hour with vehicle (DMSO) or cycloheximide (CHX), followed by inoculation with the indicated quantities of HIV-1 (A and B) or SIV (C and D) reporter viruses containing polybrene (5 μg/mL) and the respective drugs at concentrations identical to those used in the pretreatment. Four hours later, the cultures were washed and replenished with fresh media. Two days later, cells were harvested and analyzed for GFP expression by flow cytometry. Data shown are from one representative of two independent experiments.

**Supporting Method for Figure S5**

OMK (A and C) and FRhK-4 (B and D) cultures were pre-treated for one hour with the vehicle DMSO or MG132 (25μM), followed by inoculation with the indicated quantities of HIV-1 (A and B) or SIV (C and D) reporter viruses containing polybrene (5 μg/mL) and the respective drugs at concentrations identical to those used in the pretreatment. Four hours later, the cultures were washed and replenished with fresh media. Two days later, cells were harvested and analyzed for GFP expression by flow cytometry. Data shown are from one representative of two independent experiments.
